# *Vanmaneniamarmorata*, a new species of loach (Teleostei: Gastromyzontidae) from the middle Chang-Jiang Basin in Guizhou Province, south China

**DOI:** 10.3897/BDJ.9.e72432

**Published:** 2021-09-27

**Authors:** Shuqing Deng, E Zhang

**Affiliations:** 1 Institute of Hydrobiology, Chinese Academy of Sciences, Wuhan, China Institute of Hydrobiology, Chinese Academy of Sciences Wuhan China; 2 University of Chinese Academy of Sciences, Beijing, China University of Chinese Academy of Sciences Beijing China; 3 The Changjiang Civilization Museum（Wuhan Natural History Museum）, Wuhan, China The Changjiang Civilization Museum（Wuhan Natural History Museum） Wuhan China

**Keywords:** freshwater fish, new taxon, morphology, cyt b gene, phylogenetic analysis

## Abstract

**Background:**

The gastromyzontid genus *Vanmanenia* was established by Hora in 1932, based on the type species *Vanmaneniastenosoma*. The genus is a loach group adapted to running waters of streams from southern China, northern Vietnam and Laos. Currently, 19 valid species of the genus have been recognised. The northernmost distribution of the genus is the Yangtze River (= Chang-Jiang in Chinese) Basin and five species (*V.maculata*, *V.intermedia*, *V.stenosoma*, *V.pseudostriata* and *V.gymnetrus*) have been reported from the Basin.

**New information:**

*Vanmaneniamarmorata*, a new hillstream species of loach, is here described from the middle Chang-Jiang Basin in Guizhou Province, south China. It is distinguished from its congeners by having a combination of the following characters: three triangular-shaped rostral lobules; postdorsal saddles wider than interspaces; a more backwards-placed anus (the vent to anal distance 30.5–36.9% of the pelvic to anal distance); a larger gill opening with its upper extremity reaching the level of the middle of the orbit; anal-fin base length 5.6–6.4% of SL; caudal-peduncle length 11.6–12.9% of SL; prepelvic length 51.1–53.4% of SL. Its validity is also affirmed by its distinct cyt b gene sequence divergence with all sampled congeners and its monophyly recovered in a cyt b gene-based phylogenetic analysis.

## Introduction

The gastromyzontid genus *Vanmanenia* Hora, 1932, a group of loaches, occurs widely in southern China, northern Vietnam and Laos (Kottelat 2012). It has specialised rostral barbels and lips and was placed in the Crossostomini-group, a special branch in the evolution of the Gastromyzontidae (Chen 1980, Tang and Chen 2000). The genus is distinguished by having a trilobated rostral fold and two pairs of rostral barbels; lower lip with four large papillae and postlabial groove short and restricted only to mouth corner; gill opening extending ventral surface of head (Chen and Tang 2000). Currently, a total of 22 species have been identified in this genus, including three Vietnamese species whose taxonomic status is urgently needed to be confirmed: *V.nahangensis* Nguyen 2005, *V.multiloba* (Mai 1978) and *V.ventrosquamata* (Mai 1978) (Fricke et al. 2021). Three valid species (*V.crassicauda* Kottelat 2000, *V.caobangensis* Nguyen 2005 and*V.orcicampus* Kottelat 2017) are found only in the Red River Basin in Vietnam and the Mekong River Basin in Laos (Li et al. 2019, Deng and Zhang 2020). *Vanmanenia* has 16 representatives in south China where they are now known from the Yuan-Jiang (=Red River), Lixian-Jiang, Lancang-Jiang, Chang-Jiang, Zhu-Jiang, Han-Jiang, Jiulong-Jiang, Min-Jiang, Ou-Jiang, Ling-Jiang, Qiantang-Jiang and Changhua-Jiang and Wanquan-He of Hainan Island (Tang and Chen 2000, Li et al. 2019, Deng and Zhang 2020). Amongst them, there are 13 endemic Chinese species (Li et al. 2019, Deng and Zhang 2020, Fricke et al. 2021). The northernmost distribution of *Vanmanenia* is the Yangtze River (= Chang-Jiang in Chinese) Basin in which five species have been presently identified: *V.maculata*, *V.gymnetrus*, *V.intermedia*, *V.stenosoma* and *V.pseudostriata* (Yi et al. 2014, Li et al. 2019, Zhang et al. 2019, Deng and Zhang 2020, Wang and Zhang 2021). All specimens of *Vanmanenia* from this Basin had been reported as *V.pingchowensis* (Ding 1994, Zhu 1995, Chen and Tang 2000, Tang and Chen 2000) until 2014 when Yi et al. (2014) described *V.maculata* from the Qing-Jiang and Li-Shui (in the middle Chang-Jiang Basin). Later, Zhang et al. (2019) named a new species *V.pseudostriata*, based on specimens previously misidentified by Zhou et al. (2010) as *V.pingchowensis* from the Zhangjiu-He, a stream tributary to the main stem of the Jinsha-Jiang (in the upper reaches of the Chang-Jiang Basin) in Luquan County, Yunnan Province. Recently, Deng and Zhang (2020) revalidated *V.intermedia* from the synonym of *V.pingchowensis*, concluding that the former is present in the Qingshui-Jiang of the upper Yuan-Jiang of the middle Chang-Jiang Basin and the Hongshui-He of the middle Zhu-Jiang Basin. Wang and Zhang (2021) also documented *V.stenosoma* and *V.gymnetrus* from the Gan-Jiang, an effluent of Lake Poyang (in the lower Chang-Jiang Basin).

A sixth species of *Vanmanenia* from the middle Chang-Jiang Basin in Guizhou Province, south China is the one described in the present study. Field surveys of fishes conducted during June 2017 into the Yuan-Jiang, an effluent of Lake Dongting, yielded many specimens referrable to *Vanmanenia*. In comparing these specimens, they turned out to be two distinct species. The one is represented by *V.intermedia* and the other does not conform to any currently-identified congeneric Chinese species, therefore representing an unnamed species. The specific status of this unnamed species, here described as *V.marmorata*, is further affirmed by molecular phylogenetic analysis.

## Materials and methods

All measurements were taken point-to-point with a dial caliper and recorded to the nearest 0.1 mm. Morphometric data (see Table 1) and meristic counts were made on the left side of individuals when possible, following Yi et al. (2014). Prepectoral, prepelvic, predorsal and pre-anal lengths were measured from the tip of snout to the pectoral-, pelvic-, dorsal- and anal-fin origins, respectively. Caudal-peduncle length was taken from anal-fin insertion to the last vertebra. Morphometric measurements were expressed as percentages of standard length (SL) or of head length (HL). The numbers of fin rays were counted under a stereoscopic microscope. The terms utilised in this study for description of mouth part soft-tissue structures and their definitions also follow Yi et al. (2014). Data used here for *V.gymnetrus*, *V.intermedia*, *V.maculata* and*V.pseudostriata* come from Chen (1980), Deng and Zhang (2020),Yi et al. (2014) and Zhang et al. (2019), respectively. Specimens examined are deposited in the Museum of Aquatic Organisms at the Institute of Hydrobiology (IHB), Chinese Academy of Sciences, Wuhan City, Hubei Province, China (Suppl. material 1). The suffixes -Jiang, -Shui and -He mean river or stream in Mandarin Chinese.

The genomic DNA was extracted from the right-side pelvic-fin tip and mitochondrial cytochrome b (cyt b) gene was selected for amplification and sequencing. The primers and the PCR reaction followed Xiao et al. (2001) and Deng and Zhang (2020), respectively. Amplified products were subsequently purified and utilised for sequencing by a commercial sequencing company. The obtained sequences were spliced using Seqman from DNASTAR's Lasergene (Burland 2000) and then checked by utilising BLAST analysis in GenBank database. After confirming, the targeted sequences were submitted to GenBank database.

We sequenced cyt b gene of *V.stenosoma*, *V.homalocephala*, *V.hainanensis*, *V.caldwelli* and *V.marmorata* and retrieved the cyt b gene sequences for other species of *Vanmanenia* from GenBank (Table 2). *Pseudogastromyzontungpeiensis* and *Erromyzonkalotaenia* were selected as outgroups. All sequences were aligned by MAFFT 7.0 (Katoh and Standley 2013) in PhyloSuite (Zhang et al. 2020). The genetic distances (p-distance with 1,000 bootstrap) of the two sequences between taxa were calculated by utilising MEGA 7.0 (Kumar et al. 2016). The best substitution model (GTR+F+I+G4) was selected in ModelFinder (Kalyaanamoorthy et al. 2017) by Akaike’s Information Criterion (AIC). The phylogenetic trees were inferred using Bayesian Inference (BI) and Maximum Likelihood (ML) approaches. Bayesian analyses was conducted using MrBayes (Ronquist et al. 2012). Four simultaneous Monte Carlo Markov chains run for 1 million generations, with sampling one tree per 100 replicates for each run and the first quarter of the trees were discarded as burn-in; the remaining trees from two independent runs were used to construct a consensus tree. The ML analyses were conducted using IQ-TREE (Nguyen et al. 2015) with a total of 10,000 bootstrap replicates performed.

## Data resources

All the sequences in this study were retrieved from GenBank and the accession numbers of the newly-determined sequences in this study are shown in Table 2.

## Taxon treatments

### Vanmanenia marmorata

Deng & Zhang 2021 sp. n.

E60D1972-D6BD-52CE-8EC5-1514829F7155

urn:lsid:zoobank.org:act:1C9F4415-847D-4124-8D64-575560F3056A

#### Materials

- **Type status:**Holotype. **Occurrence:** recordNumber: IHB2017060069; recordedBy: Changting An; individualCount: 1; lifeStage: adult; **Taxon:** scientificName: *Vanmaneniamarmorata*; kingdom: Animalia; phylum: Chordata; class: Actinopterygii; order: Cypriniformes; family: Gastromyzontidae; genus: Vanmanenia; **Location:** waterBody: the Chang-Jiang basin; country: China; stateProvince: Guizhou Province; county: Jiangkou County; locality: the Chenshui River, a tributary of the Yuan-Jiang drainage; verbatimElevation: 866.1 m a.s.l.; verbatimCoordinates: 27°52′16.94″N, 108°37'15.6''E; georeferenceSources: Google Earth; **Identification:** identifiedBy: Shuqing Deng; dateIdentified: 2017-9-20; **Event:** eventDate: 2017-6-22; **Record Level:** collectionCode: fish; basisOfRecord: Preserved Specimen
- **Type status:**Paratype. **Occurrence:** recordNumber: IHB2017060068, 5189–5191; recordedBy: Changting An; individualCount: 4; **Taxon:** scientificName: *Vanmaneniamarmorata*; kingdom: Animalia; phylum: Chordata; class: Actinopterygii; order: Cypriniformes; family: Gastromyzontidae; genus: Vanmanenia; **Location:** waterBody: the Chang-Jiang basin; country: China; stateProvince: Guizhou Province; county: Jiangkou County; locality: the Chenshui River, a tributary of the Yuan-Jiang drainage; verbatimElevation: 866.1 m a.s.l.; verbatimCoordinates: 27°52′16.94″N, 108°37'15.6''E; georeferenceSources: Google Earth; **Identification:** identifiedBy: Shuqing Deng; dateIdentified: 2017-9-20; **Event:** eventDate: 2017-6-22; **Record Level:** collectionCode: fish; basisOfRecord: Preserved Specimen

#### Description

**Figure 1.**
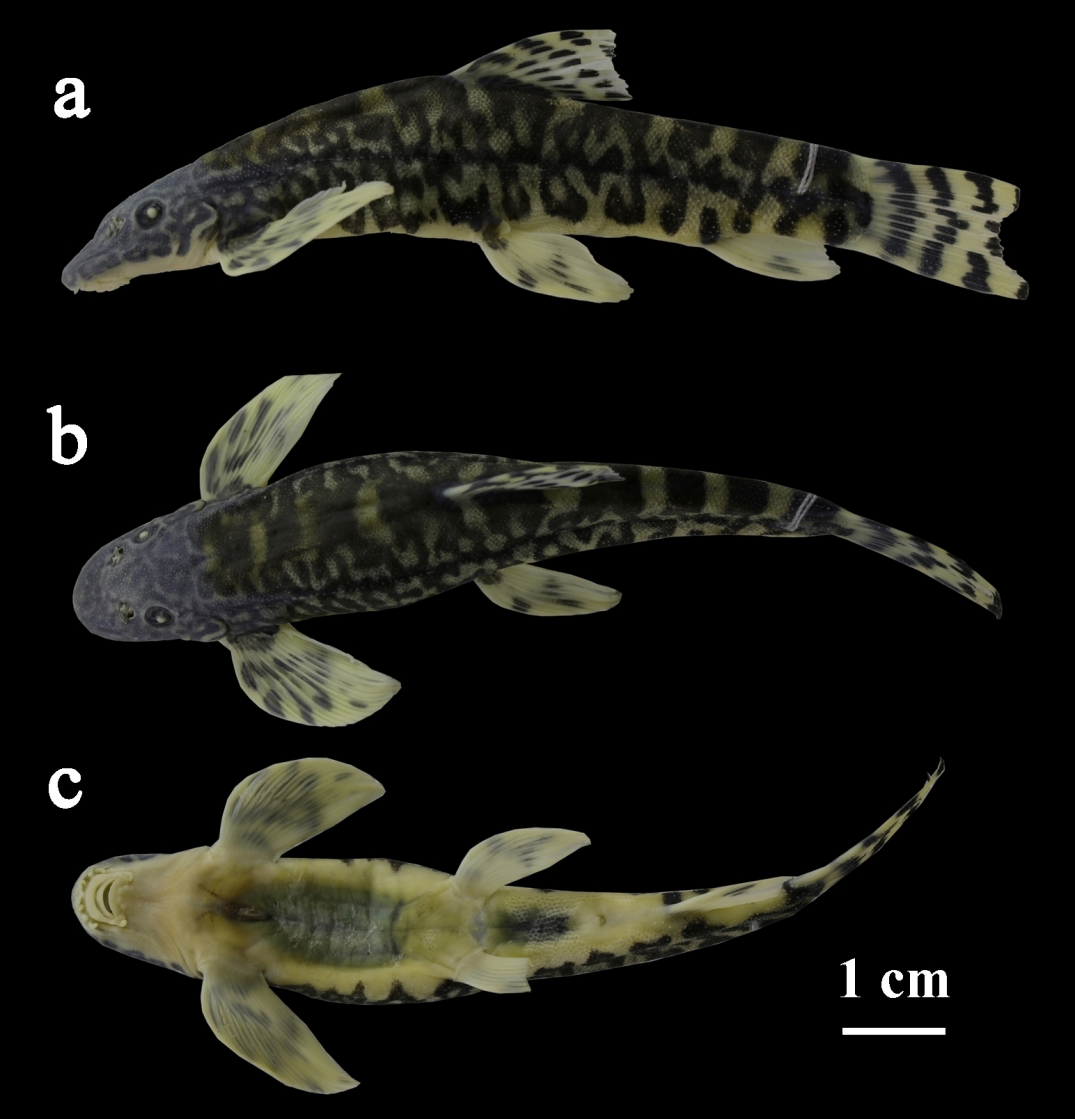
*Vanmaneniamarmorata*, IHB2017060069, holotype, 68.8 mm SL; Guizhou Province: Jiangkou County. Lateral (a),dorsal (b) and ventral(c) views of body.

Morphometric measurements for specimens examined are given in Table 1. See Fig. 1a-c for lateral, dorsal and ventral view of body.

**Figure 2.**
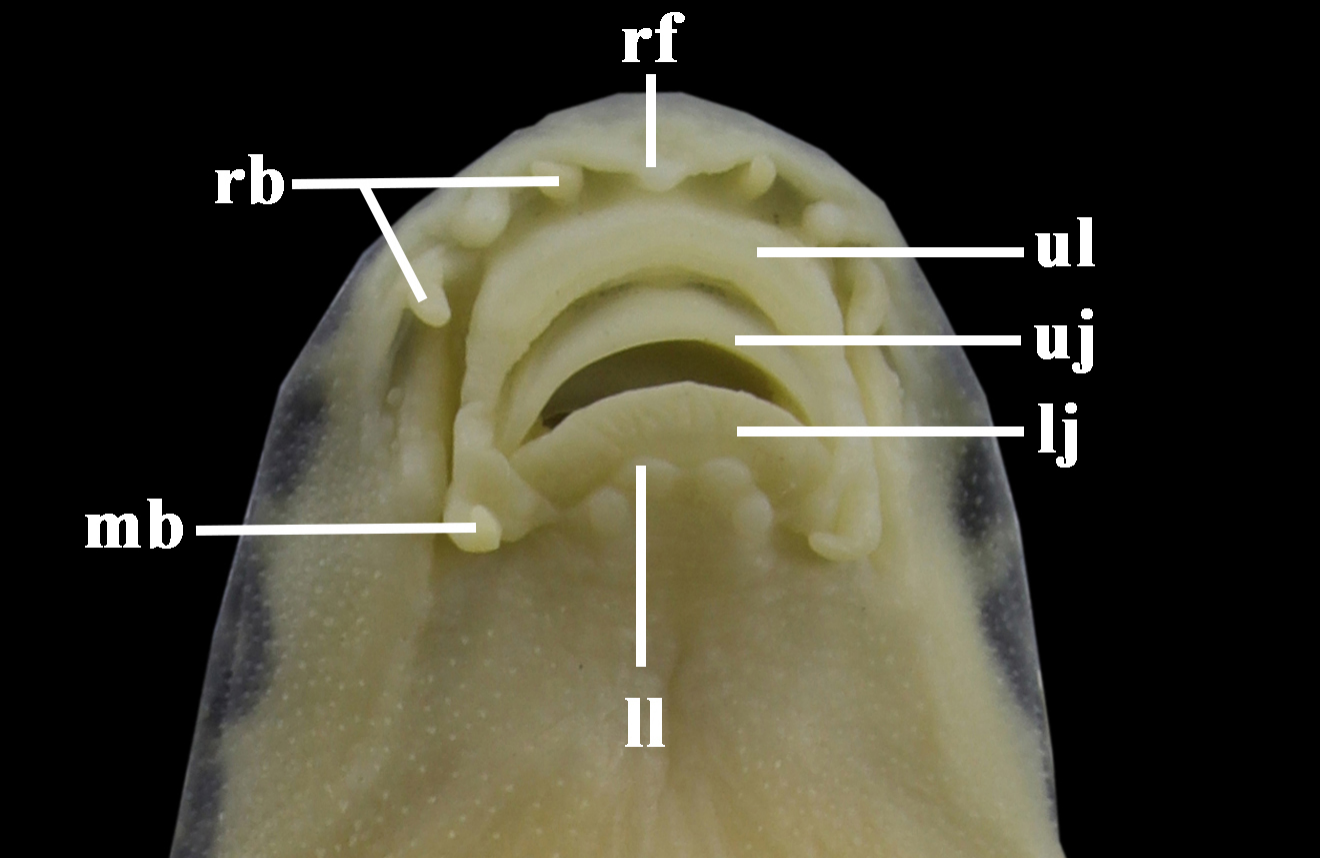
Ventral view of mouth of *Vanmaneniamarmorata*. IHB2017060069, holotype, 68.8 mm SL; Guizhou Province: Jiangkou County. lj: lower jaw; mb: maxillary barbel; rb: rostral barbel; rf: rostral fold; uj: upper jaw; ul: upper lip; ll: lower lip.

Body elongate and slightly compressed, with greatest depth at dorsal-fin origin and least depth at caudal-fin base. Dorsal profile of head rising abruptly before, then increasing evenly towards dorsal-fin origin, from there, to caudal-fin base decreasing gradually. Lower surface of head straight and oblique; ventral profile of body from pectoral-fin insertion to anal-fin origin slightly concave, oblique along anal-fin base and straight in caudal peduncle. Head moderately depressed, slightly longer than wide and wider than high, with slightly broad and convex interorbital space. Snout slightly rounded in dorsal view. Eyes small, dorsolateral in posterior half of head. Anterior and posterior nostrils separated and short flap on anterior ones. Mouth small, inferior and arched. Rostral fold divided into three triangular-shaped lobules, median one slightly wider than two lateral ones; tips of these lobules in barbel-like form, but not modified into secondary rostral barbels (Fig. 2). One pair of maxillary barbels at corners of mouth; two pairs of rostral barbels in deep groove present between rostral fold and upper lip. Upper lip pendulous and connected with lower lip around corners of mouth by papillated flap; lower lip with four large papillae, two median ones more anteriorly placed and usually larger than the two lateral ones; postlabial groove short and restricted only to mouth corner. Upper and lower jaws bearing thick, with flexible horny sheaths on flanks. Gill opening reaching ventral surface of head, with its upper extremity reaching the level of the middle of the orbit. Body scaled, but absent on head; scales minute, lateral-line complete with 78–90 perforated scales. Anus placed significantly nearer anal-fin origin than pelvic-fin origin.

Dorsal fin with 3 unbranched and 7–8 branched rays, nearly as long as head; dorsal-fin origin almost located halfway between caudal-fin base and snout tip; distal margin slightly concave. Pectoral fin with 1 unbranched and 13–14 branched rays, longer than head; inserted slightly behind lower extremity of gill-opening; tip of adpressed rays not reaching pelvic-fin insertion; distal margin convex. Pelvic fin with 1 unbranched and 8 branched rays, inserted slightly closer to caudal-fin base than to snout tip or midway between pectoral-fin insertion and anal-fin origin or slightly moved backwards; tip of adpressed rays surpassing anus, but far from anal-fin origin; distal margin pointed; axillary lobe present at pelvic-fin base. Anal fin with 2 unbranched and 5 branched rays, last one split to base; origin closer to caudal-fin base than to pelvic-fin insertion; distal margin convex. Anus positioned closer to anal-fin origin than to posterior end of pelvic-fin base. Anal fin extending beyond ventral origin of caudal-fin procurrent rays. Caudal fin slightly forked with lower lobe slightly longer than upper one.

##### Coloration

In alcohol-stored specimens, top of head with numerous, small, irregular, black bars and blotches; yellowish on ventral surface of head and abdomen. Body with 7-9 dorsal dark black saddles. Last predorsal, subdorsal and first postdorsal saddles wider than all others. Postdorsal saddles wider than interspaces. A number of irregular black blotches on flank above lateral line, nearly connected with dorsal saddles. These black blotches extended downwards to ventral surface of body. Blotch through pelvic-fin insertion extended downwards to base of axillary lobe at pelvic-fin base. A black spot on caudal-fin base, darker than markings on flank. Dorsal fin with 3 black bands across rays. Anal fin with 1 black band. Pectoral fin with 3 irregular faint black bands across rays. Pelvic fin with up to 3 black bands. Caudal fin with 4 black bands.

#### Diagnosis

*Vanmaneniamarmorata* resembles the four species (*V.caldwelli*, *V.maculata*, *V.intermedia* and *V.stenosoma*) in having three triangular-shaped rostral lobules whose apical portions are in the barbel-like form, but not modified into secondary rostral barbels. It is distinct from these species in having postdorsal dark black saddles wider (vs. narrower) than their interspaces, further from *V.caldwelli* in having no longitudinal black stripe extending from the snout tip to the caudal-fin base along the lateral line on flank (vs. present) and a more backwards-placed anus [the vent to anal distance 30.5–36.9% (mean 34.6) vs. 60.0–70.3% (mean 68.5) of the pelvic to anal distance]; from *V.maculata* in having a dark black vermiculated mark (vs. large brown blotch; see Yi et al. 2014: Page 90, fig. 2) on the submargin of the gill cover and a more backwards-positioned anus [the vent to anal distance 30.5–36.9% (average 34.6) vs. 36.4–48.4% (average 43.0) of the pelvic to anal distance] (see Table 3); and from *V.intermedia* in having a larger gill opening with its upper extremity reaching the level of the middle of the orbit (vs. smaller, closer to the level of the lower margin of the orbit; see Deng and Zhang 2020 : Page 117: fig. 2), a shorter (vs. longer) anal-fin base [length 5.6–6.4 (mean 6.0) vs. 7.5–9.5 (mean 8.3) % of SL) and a longer (vs. shorter) caudal peduncle [length 11.6–12.9 (average 12.0) vs. 8.4–11.1 (average 9.9) % of SL]; and from *V.stenosoma* in having a longer (vs. shorter) caudal peduncle [length 11.6–12.9 (mean 12.0) vs. 9.0–11.1 (mean 10.0) % of SL] and a more forwards-positioned pelvic fin [prepelvic length 51.1–53.4 (mean 51.7) vs. 54.7–59.2 (mean 57.2) % SL].

#### Etymology

The specific epithet is from the Latin word *marmor* referring to the unique body colouration of irregular marbled markings.

#### Distribution

**Figure 3.**
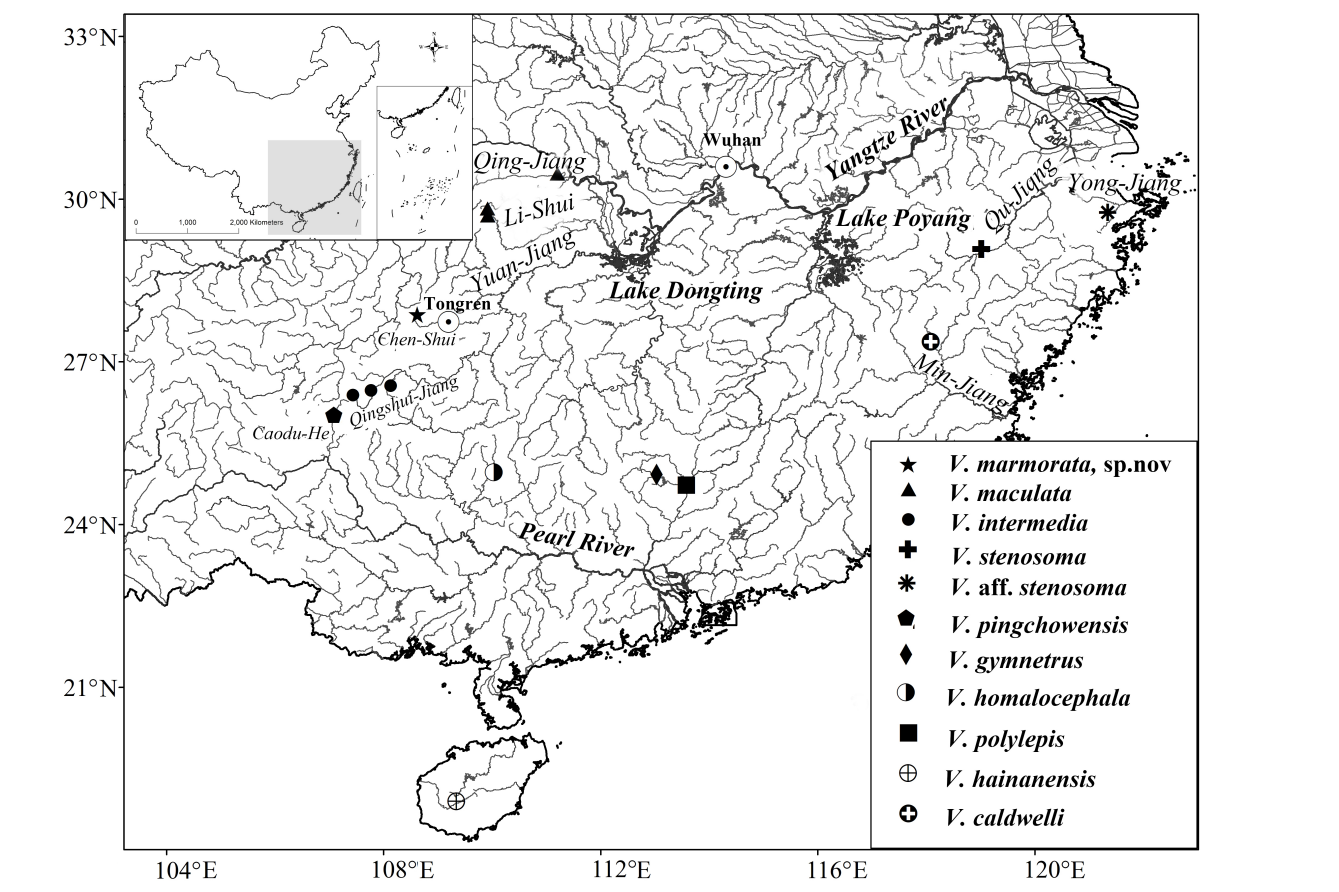
Map showing collection localities of 11 species of *Vanmanenia* involved in molecular biology studies. The names of rivers and lakes are italicised and two cities (Wuhan and Tongren) highlighted.

**Figure 4.**
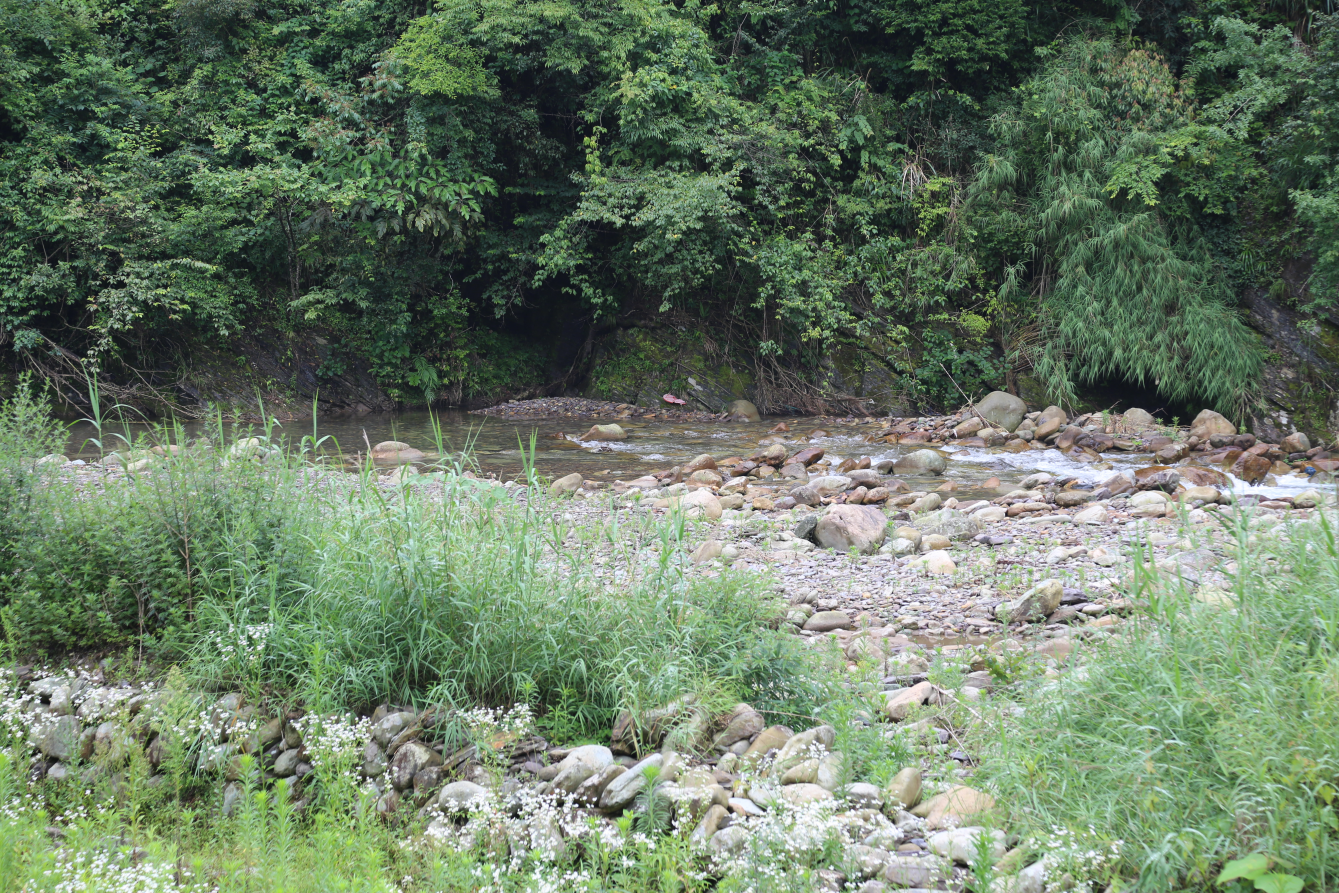
Locality of *Vanmaneniamarmorata*, Jiangkou County, Guizhou Province.

This new species is presently known from the upper reaches of the Chen-Shui, a stream tributary to the Yuan-Jiang of the Dongting Lake system in the middle Chang-Jiang Basin, at Jiangkou County, Guizhou Province, south China (Fig. 3). It inhabits fast-flowing waters with a gravelly and pebbly substrate (Fig. 4). Co-existing species are *Discogobioyunnanensis* (Regan, 1907), *Onychostomabarbatum* (Lin, 1931) and *Rhinogobiuscliffordpopei* (Nichols, 1925).

## Identification Keys

### Key to six species of *Vanmanenia* in the Yangtze River

**Table:** 

1	Secondary rostral barbels present	* V. gymnetrus *
–	Secondary rostral barbels absent	2
2	Rostral lobules rounded; 9-10 black bars on flank	* V. pseudostriata *
–	Rostral lobules triangular-shaped; no black bars on flank	3
3	Gill opening smaller, with its upper extremity aligned with lower margin of eye	* V. intermedia *
–	Gill opening larger, with its upper extremity reaching level of middle of eye	4
4	4 Post-dorsal saddles across dorsum wider than interspaces	* V. marmorata *
–	Post-dorsal saddles across dorsum narrower than interspaces	5
5	Caudal peduncle stout, deeper than long; a dark black bar present on caudal-fin base	* V. stenosoma *
–	Caudal peduncle slender, longer than deep; no black bar on caudal-fin base	* V. maculata *

## Analysis

**Sequence variation and molecular phylogeny**

Thirty-one cyt b gene sequences of *Vanmanenia* were used for phylogenetic analysis. After alignment and trimming, 1095 bp (base pairs) of the cyt b gene were obtained. There were 735 conserved sites, 360 variable sites, 268 parsimony informative sites and 92 singleton sites. The mean frequency of four nucleotides was A=25.9%, T=29.3%, C= 29.6% and G= 15.2%; the base composition was A–T rich (55.2%).

**Figure 5.**
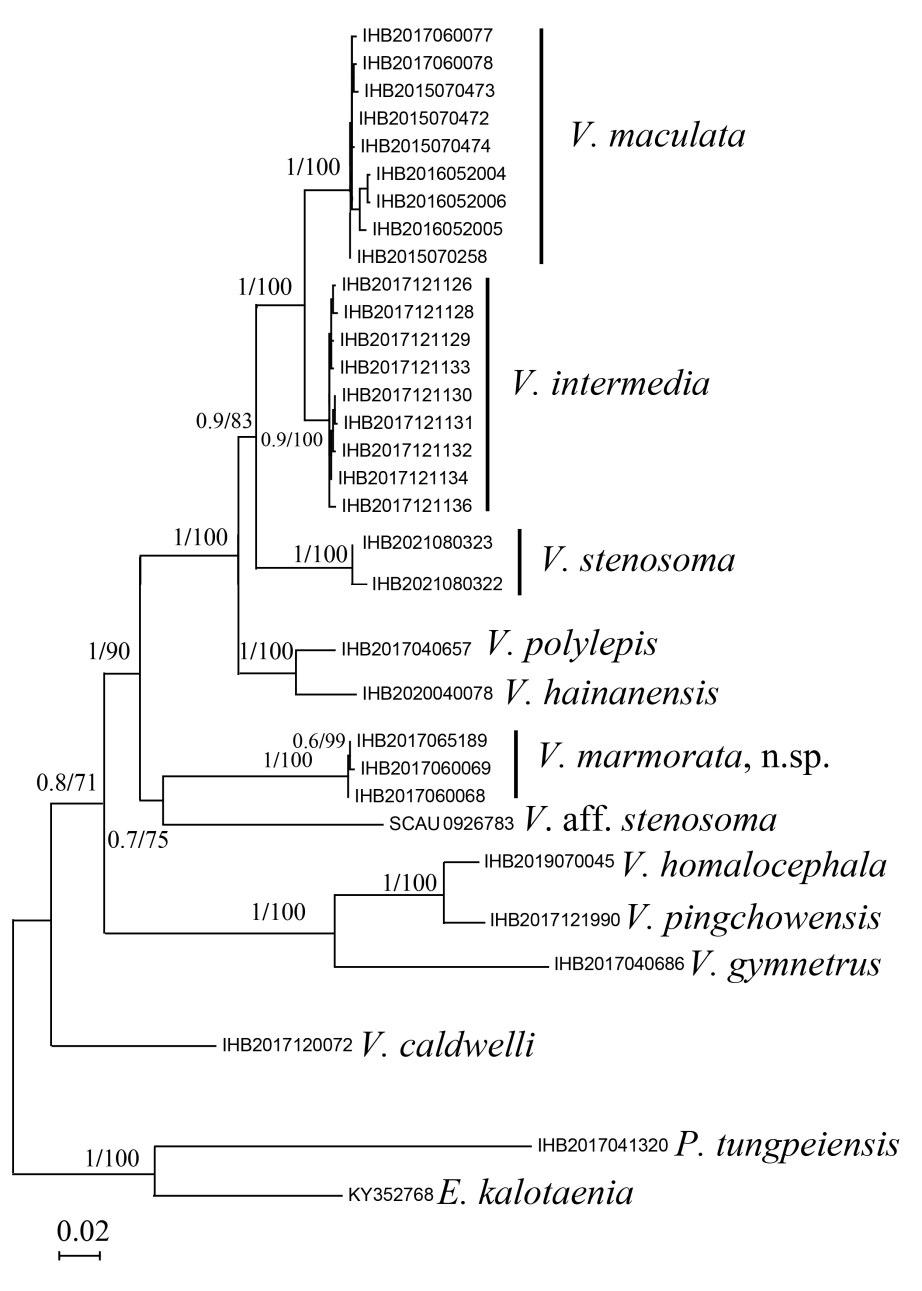
Phylogenetic tree reconstructed using Bayesian method (BI) and Maximum Likelihood (ML), based on cyt b gene. Values at the nodes correspond to the support values for BI/ML methods.

The two analysis methods (BI and ML) showed an identical topology (Fig. 5). From the tree topology, samples of this new species constituted a strongly-supported (100% posterior probabilities and 100% bootstrap values) independent lineage that was distantly related to the lineage formed by topotypical samples of *V.stenosoma* from the Yong-Jiang, but nested with the lineage constituted by the sample (SCAU0926783), under the name of *V.stenosoma*, from Qu-Jiang into a clade (hereafter called V.aff.stenosoma) sister to the clade made of by samples from the following five species: *V.maculata*, *V.intermedia*, *V.stenosoma*, *V.hainanensis* and *V.polylepis*.

*Vanmaneniamarmorata* had the minimum genetic distance of 11.3% with *V.stenosoma*and the maximum one of 15.4% with *V.gymnetrus* (Table 4). The mean genetic distance of the new species with all sampled Chinese congeneric species was 13.0%, far greater than the minimum ones (2.6%), here detected between *V.pingchowensis* and *V.homalocephala*. The new species had a significant sequence divergence from *V.maculata* and *V.pingchowensis* (12.2–15.2%). The intraspecific genetic divergence of *V.marmorata* was 0.2%.

## Discussion

The new species can be assigned to the group characterised by having three triangular-shaped rostral lobules whose apical parts are in the barbel-like form and connected with the rostral lobule, but not modified into secondary rostral barbels, based on the grouping of Chinese species of *Vanmanenia* by Yi (2014). Four species are, so far, identified in this group, namely *V.intermedia*, *V.maculata*, *V.stenosoma* and *V.caldwelli* (see the diagnosis for detailed differences of this new species with these four species).

*Vanmaneniamarmorata* is presently known only from the Yuan-Jiang of the middle Chang-Jiang Basin. In addition to *V.intermedia*, *V.maculata* and *V.stenosoma*, there are two other congeneric species *V.pseudostriata* and *V.gymnetrus* also found in the upper and lower Chang-Jiang Basin, respectively. Within *Vanmanenia*, *V.pseudostriata* was assigned to either the barred group defined by Li et al. (2019) as having vertical black bars on flank and spotless paired fins or the first group by Yi (2014) as having three rounded rostral lobules. These three characters can separate the new species from *V.pseudostriata* with vermiculated marks on the flank, up to three bands across the rays of paired fins and three triangular-shaped rostral lobules. The new species is further distinct from *V.pseudostriata* in possessing a more backwards-set anus (closer to the anal-fin origin than to the pelvic-fin insertion vs. midway between the pelvic-fin insertion and anal-fin origin) and less lateral-line scales (78−90 vs. 95−100). *Vanmaneniagymnetrus* was transferred to the fourth group, defined by Yi (2014) as having multiple barbel-like forms derived from the apical parts of the rostral lobules, as well as secondary rostral barbels. This group is the same as ‘the barbelled rostral fold group’ defined by Li et al. (2019) as having three rostral lobes modified into papillae and secondary barbels. *V.gymnetrus* is further distinct from *V.marmorata* in having more lateral-line scales (92−104 vs. 78−90), a slender (vs. stout) caudal peduncle (depth 0.63−0.67 vs. 0.72−1.01% of length) and a narrower (vs. wider) interorbital space (width 35.7−37.0 vs. 37.3−41.0% of HL).

The validity of *V.marmorata* is corroborated by its remarkable genetic divergence from sampled congeners (Table 4) and its monophyly recovered in the phylogenetic analysis, based on the cyt b gene (Fig. 5). The new species had a 0.2% intraspecific genetic divergence and a significant genetic divergence with sampled congeneric Chinese species (11.3–15.4%), far greater than the minimum calculated here between *V.pingchowensis* and *V.homalocephala* (2.6%). The topology of the phylogenetic tree also showed that the new species was sister to V.aff.stenosoma, represented by the sample (SCAU0926783) previously misidentified as *V.stenosoma* from the Qu-Jiang. The type locality of *V.stenosoma* is Ningbo, southern Zhejiang Province, in the Yong-Jiang, a coastal river flowing into the South China Sea. Two topological samples of *V.stenosoma* were highly supported to unite with samples of the paired species (*V.maculata* and *V.intermedia*) into an lineage, and distantly related to the sample (SCAU0926783), under the name of *V.aff.stenosoma*, from the Qu-Jiang. This sample (SCAU0926783) might represent an undescribed species distinct from this new species, given a clear barcode gap between the paired species (11.9%).

## Supplementary Material

08B46942-FF59-591C-B048-A3C993AD6316 10.3897/BDJ.9.e72432.suppl1 Supplementary material 1 Table S1. Material examined of *Vanmanenia* species from China Data type Specimen list Brief description Comparative material File: oo_585070.docx https://binary.pensoft.net/file/585070 Shuqing Deng and E Zhang

XML Treatment for Vanmanenia marmorata

## Figures and Tables

**Table 1.** Morphometric measurements for *Vanmaneniamarmorata*.

	Holotype	Holotype + Paratypes
		Range (mean±SD)
SL(mm)	68.8	36.2–71.9
Morphometric measurements		
% of SL		
Body depth	20.3	14.9–20.3（17.5±1.89）
Head length	21.3	19.0–21.9（20.6±1.19）
Head width	18.3	16.4–18.3（17.4±0.70）
Head depth	12.1	10.5–12.1（11.5±0.67）
Caudal-peduncle length	11.6	11.6–12.9（12.0±0.52）
Caudal-peduncle depth	11.8	9.3–11.8（11.0±0.98）
Dorsal-fin length	22.6	20.5–22.6（21.3±0.81）
Pectoral-fin length	26.2	25.1–26.2（25.8±0.44）
Pelvic-fin length	20.7	19.5–20.8（20.4±0.57）
Anal-fin length	18.5	16.5–19.2（17.7±1.11）
Dorsal-fin base length	13.3	11.6–13.3（12.3±0.71）
Pectoral-fin base length	8.5	7.4–8.5（8.2±0.47）
Pelvic-fin base length	6.0	4.8–6.0（5.0±0.68）
Anal-fin base length	6.3	5.6–6.4（6.0±0.31）
Prepectoral length	18.2	18.2–20.2（19.2±0.75）
Predorsal length	50.1	48.5–52.2（50.2±1.31）
Prepelvic length	51.7	51.1–53.4（51.7±0.96）
Pre-anal length	80.5	79.0–83.7（80.8±1.72）
Pectoral- to pelvic-fin origin	31.7	31.7–35.7（32.8±1.64）
Pelvic- to anal-fin origin	29.7	27.8–29.7（28.9±0.70）
% of HL		
Snout length	48.6	46.7–53.3（49.1±2.58）
Mouth width	32.4	28.4–32.9（30.9±1.93）
Eye diameter	20.9	20.9–25.2（23.0±1.62）
Interorbital width	38.8	37.0–41.0（38.7±1.50）
of caudal-peduncle length		
Width of caudal peduncle	1.01	0.72–1.01（0.92±0.10）
% of pelvic to anal distance		
Vent to anal-fin origin	30.5	30.5–36.9（34.6±2.57）
Predorsal/prepelvic length (%)	96.8	94.9–98.3（97.1±1.32）

**Table 2.** The species used in this study with their GenBank accession number for the mitochondrial cyt b gene sequences.

**Species**	**Specimen voucher**	**Sampling localities**	**GenBank accession number**	**Source**
* V. marmorata *	IHB2017060068	China: Guizhou: Jiangkou	OK040742	This study
* V. marmorata *	IHB2017060069	China: Guizhou: Jiangkou	OK040743	This study
* V. marmorata *	IHB2017065189	China: Guizhou: Jiangkou	OK040744	This study
* V. maculata *	IHB2017060077	China: Hunan: Sangzhi	MT561194	GenBank
* V. maculata *	IHB2017060078	China: Hunan: Sangzhi	MT561195	GenBank
* V. maculata *	IHB2015070258	China: Hunan: Sangzhi	MT561198	GenBank
* V. maculata *	IHB2015070472	China: Hubei: Hefeng	MT561202	GenBank
* V. maculata *	IHB2015070473	China: Hubei: Hefeng	MT561203	GenBank
* V. maculata *	IHB2015070474	China: Hubei: Hefeng	MT561204	GenBank
* V. maculata *	IHB2016052004	China: Hubei: Changyang	MT561206	GenBank
* V. maculata *	IHB2016052005	China: Hubei: Changyang	MT561207	GenBank
* V. maculata *	IHB2016052006	China: Hubei: Changyang	MT561208	GenBank
* V. intermedia *	IHB2017121126	China: Guizhou: Kaili	MT561252	GenBank
* V. intermedia *	IHB2017121128	China: Guizhou: Kaili	MT561253	GenBank
* V. intermedia *	IHB2017121129	China: Guizhou: Kaili	MT561254	GenBank
* V. intermedia *	IHB2017121130	China: Guizhou: Kaili	MT561255	GenBank
* V. intermedia *	IHB2017121131	China: Guizhou: Kaili	MT561256	GenBank
* V. intermedia *	IHB2017121132	China: Guizhou: Kaili	MT561257	GenBank
* V. intermedia *	IHB2017121133	China: Guizhou: Duyun	MT561258	GenBank
* V. intermedia *	IHB2017121134	China: Guizhou: Duyun	MT561259	GenBank
* V. intermedia *	IHB2017121136	China: Guizhou: Duyun	MT561261	GenBank
* V. gymnetrus *	IHB2017040686	China: Guizhou: Ruyuan	MT561227	GenBank
* V. homalocephala *	IHB2019070045	China: Guangxi: Yongfu	OK040741	This study
* V. polylepis *	IHB2017040657	China: Guangdong: Shaoguan	MT561219	GenBank
* V. hainanensis *	IHB2020040078	China: Hainan	OK040745	This study
* V. pingchowensis *	IHB2017121990	China: Guizhou: Pingtang	MT561242	GenBank
* V. stenosoma *	IHB2021080322	China: Zhejiang: Ningbo	OK040739	This study
* V. stenosoma *	IHB2021080323	China: Zhejiang: Ningbo	OK040740	This study
V. aff. stenosoma	SCAU 0926783	China: Zhejiang: Quzhou	KX786161	GenBank
* V. caldwelli *	IHB2017120072	China: Fujian: Nanpin	OK040746	This study
* P. tungpeiensis *	IHB2017041320	China: Jiangxi: Shicheng	MT561266	GenBank
* E. kalotaenia *	IHB20080401	China: Guangxi: Mt. Dayaoshan	KY352768	GenBank

**Table 3.** Comparisons of main characters amongst species of *V.marmorata,V.intermedia, V.maculata, V.stenosoma* and *V.pseudostriata* (Data from: a-Deng and Zhang (2020); b-Yi et al. (2014); c-Zhang et al. (2019)).

	* V. marmorata *	* V. intermedia * ^a^	* V. maculata * ^a,^	* V. stenosoma * ^a^	* V. pseudostriata * ^c^
Shape of rostral fold	Triangular	Triangular	Triangular	Triangular	Rounded
Upper extremity of gill opening	Reaching level of middle of eye	Closer to level of lower margin of eye	Reaching level of middle of eye	Reaching level of middle of eye	Closer to level oflower margin of eye
Vertical bars	Absent	Absent	Absent	Absent	9–10 regular bars
Post-dorsal saddles across dorsum	Wider than interspaces	Narrower than interspaces	Narrower than interspaces	Narrower than interspaces	Narrower than interspaces
Marks on paired-fin rays	Present	Present	Present	Present	Absent
Caudal-peduncle length	11.6–12.9(12.0±0.52)	8.4–11.1(9.9±0.90)	9.8–13.4(11.5±0.99)	9.0–11.1(10.0±0.70)	–
Prepelvic length	51.1–53.4(51.7±0.96)	52.7–57.1(55.3±1.30)	52.0–57.8(54.7±1.48)	54.7–59.2(57.2±1.47)	50–52.6
Anal-fin base length % of SL	5.6–6.4(6.0±0.30)	7.5–9.5(8.3±0.65)	5.2–7.2(6.2±0.45)	5.1–6.4(5.6±0.43)	–
Anus to anal-fin distance % of pelvic to anal distance	30.5–36.9(34.6±2.60)	34.7–46.1(41.2±3.51)	36.4–48.4 (43.0±3.37)	29.8–37.1(34.3±1.90)	–
Predorsal length % of prepelvic length	94.9–98.3(97.1±1.32)	89.0–93.2(91.0±1.40)	93.7–98.5(95.2±1.10)	89.4–94.9(90.8±1.40)	–
Lateral-line scales	78–90	70–88	72–95	90–100	95–100

**Table 4.** Genetic distances of cyt b computed by MEGA amongst 11 species of *Vanmanenia*.

	1	2	3	4	5	6	7	8	9	10
1. *V.marmorata*										
2. *V.maculata*	0.122									
3. *V.intermedia*	0.117	0.035								
4. *V.stenosoma*	0.113	0.074	0.066							
5. V.aff.stenosoma	0.119	0.128	0.121	0.127						
6. *V.pingchowensis*	0.152	0.147	0.140	0.144	0.154					
7. *V.gymnetrus*	0.154	0.163	0.149	0.148	0.180	0.107				
8. *V.homalocephala*	0.153	0.147	0.141	0.150	0.154	0.026	0.107			
9. *V.polylepis*	0.120	0.069	0.065	0.075	0.128	0.157	0.163	0.157		
10. *V.hainanensis*	0.124	0.079	0.075	0.080	0.122	0.157	0.163	0.159	0.042	
11. *V.caldwelli*	0.126	0.128	0.123	0.121	0.141	0.155	0.163	0.155	0.133	0.128
