# Supplementary material for: Vanmaneniamarmorata, a new species of loach (Teleostei: Gastromyzontidae) from the middle Chang-Jiang Basin in Guizhou Province, south China
Source: Biodivers Data J. 2021 Sep 27;9:e72432. doi: 10.3897/BDJ.9.e72432 (PMC8490344; doi:10.3897/BDJ.9.e72432)
Supplement: Supplementary material 1 — Table S1. Material examined of Vanmanenia species from China [file bdj-09-e72432-s001.docx]

**Supplementary material 1**

**Table S1. Material examined of *Vanmanenia* species from China**

Authors: Shuqing Deng and E Zhang

Data type: Specimen list

**Table S1. Material examined of *Vanmanenia* species from China**

| Species | Catalog Number | Number of  Specimens | Standard  Length (mm) | Locality |
| --- | --- | --- | --- | --- |
| *V. caldwelli* | IHB 54V0374–0375, 54V0301–0307 | 9 | 40.2–70.5 | Chong’an, Fujian |
| *V. maculata* | IHB 2016054965, 2006064912–4916 | 6 | 54.5–78.1 | Jianshi, Hubei |
|  | IHB 2016044949–4958, 2016054959, 2016054961, 2017074901–4910 | 22 | 44.9–83.9 | Yichang, Hubei |
| *V. gymnetrus* | IHB 825245 | 1 | 88.2 | Chong’an, Fujian |
|  | Uncatalogued specimens | 2 | 70.0–76.5 | Longyan, Fujian |
| *V. hainanensis* | IHB 76V9142, 76V9168, 76V9173–79, 76V9181, 76V9183, 76V9185, 76V9188, 76V9090–9091 | 15 | 53.2–86.9 | Qiongzhong, Hainan |
|  | IHB76V9169–9172, 76V9625, 76V9180, 76V9182, 76V9184, 76V9189, 76V9192 | 10 | 43.2–85.4 | Qiongzhong, Hainan |
| *V. homalocephala* | IHB 2017122234–2238 | 5 | 72.8–83.9 | Jinxiu, Guangxi |
| *V. lineata* | Uncatalogued specimens | 2 | 40.1–58.7 | Dayao Mountain, Guangxi |
| *V. pingchowensis* | IHB 2017121990–1992, 2017122220–2225 | 9 | 67.5–90.4 | Pingtang, Guizhou |
| *V. stenosoma* | IHB 74 VII3870–3872 | 3 | 48.2–59.3 | Fenghua, Zhejiang |
|  | IHB 74VII3368–3374, 74VII3376–3381 | 13 | 57.5–70.9 | Tiantai, Zhejiang |
| *V. xinyiensis* | IHB 77VII074–075, 77VII077–079, 77VII081, 77VII090, 77VII475 | 8 | 58.4–96.3 | Xinyi, Guangdong |
